# Leptospirosis in the Caribbean: a literature review

**DOI:** 10.26633/RPSP.2017.166

**Published:** 2017-12-19

**Authors:** Abena Peters, Alexandra Vokaty, Richard Portch, Yitades Gebre

**Affiliations:** 1 Pan American Health Organization (PAHO) Country Office, Port of Spain, Trinidad and Tobago. Pan American Health Organization (PAHO) Country Office, Port of Spain, Trinidad and Tobago.; 2 PAHO Country Office Paramaribo Suriname PAHO Country Office, Paramaribo, Suriname.

**Keywords:** Leptospirosis, animal diseases, disease vectors, review, Caribbean Region, Leptospirosis, enfermedades de los animales, vectores de enfermedades, revisión, Región del Caribe, Leptospirose, doenças dos animais, vetores de doenças, revisão, Região do Caribe

## Abstract

**Objective.:**

*To describe leptospirosis epidemiology, seroprevalence, and serovars among humans and animals in the Caribbean from 1979 – 2013*.

**Methods.:**

*A retrospective study of the literature was performed on the general epidemiology, historical records, and geographical locations of leptospirosis outbreaks and cases in the Caribbean from 1979 — 2013. The primary sources of information were identified with PubMed Central, Google Scholar, CAREC, CaribVET, and The School of Veterinary Medicine at the University of the West Indies. Search terms used were: “human leptospirosis,” “animal leptospirosis,” “serovars,” “livestock,” “seroprevalence,” “Caribbean countries,” “risk factors,” “confirmed cases,” “suspected cases,” “MAT,” and “ELISA.” Confirmed and suspected cases of human and animal leptospirosis were identified through laboratory analysis*.

**Results.:**

*Most cases of leptospirosis occurred during the rainy season (June — December) and had a positive correlation with flood conditions. The disease was more prevalent in males than females due to behavioral and occupational exposure. The highest incidence rates of human leptospirosis were recorded in Barbados, Trinidad and Tobago, and Jamaica. In animals, leptospirosis has been found in rodents, livestock, and dogs in many Caribbean countries. Inadequate active surveillance and misdiagnosis of human leptospirosis has contributed to under-reporting of the disease*.

**Conclusion.:**

*This review highlights the epidemiology and distribution of leptospirosis in the Caribbean. Prevalence rates and serovars vary greatly among the countries. Leptospirosis poses a significant health risk for humans and animals in the Caribbean and requires a “One Health” multisectoral approach to reduce incidence rates and protect at-risk individuals. Increased laboratory capacity to identify leptospirosis cases is required, along with awareness campaigns for both the public and animal and human health professionals*.

## RESUMEN

**Objetivo.:**

Describir las características epidemiológicas de la leptospirosis, su seroprevalencia y serovariedades en seres humanos y animales en el Caribe desde 1979 hasta el 2013.

**Métodos.:**

Se realizó un estudio retrospectivo de la bibliografía en relación con las características epidemiológicas generales, los registros históricos y las ubicaciones geográficas de los brotes y casos de leptospirosis en el Caribe desde 1979 hasta el 2013. Se determinaron las fuentes primarias de información con PubMed central, Google Scholar, CAREC, CaribVET y la Escuela de Medicina Veterinaria de la Universidad de las Indias Occidentales. Los términos de búsqueda que se usaron en inglés fueron los equivalentes de “leptospirosis humana”, “leptospirosis animal”, “serovariedades”, “ganado”, “seroprevalencia”, “países del Caribe”, “factores de riesgo”, “casos confirmados”, “presuntos casos”, “MAT” y “ELISA”. Se determinaron los casos confirmados y presuntos de leptospirosis humana y animal mediante el análisis de laboratorio.

**Resultados.:**

La mayor parte de los casos de leptospirosis se dieron durante la temporada de lluvias (junio a diciembre) y tuvieron una correlación positiva con las inundaciones. La enfermedad fue más prevalente en los hombres que en las mujeres debido a la exposición laboral y por los comportamientos. Las tasas de incidencia más altas de leptospirosis humana se registraron en Barbados, Trinidad y Tabago y Jamaica. En el caso de los animales, la leptospirosis se ha detectado en los roedores, el ganado y los perros de muchos países del Caribe. La vigilancia activa insuficiente y el diagnóstico erróneo de la leptospirosis humana han contribuido a que haya una subnotificación de los casos de esta enfermedad.

**Conclusiones.:**

En esta revisión se ponen de relieve las características epidemiológicas y la distribución de la leptospirosis en el Caribe. Las tasas de prevalencia y las serovariedades varían enormemente de un país a otro. La leptospirosis supone un riesgo considerable para la salud de los seres humanos y los animales en el Caribe y requiere un enfoque multisectorial de “Una Salud” para reducir la incidencia y proteger a las personas en riesgo. Se necesita una mayor capacidad en materia de laboratorios para detectar los casos de leptospirosis, junto con campañas de sensibilización tanto del público como de los profesionales de la sanidad animal y la salud humana.

## RESUMO

**Objetivo.:**

Descrever o perfil epidemiológico, a soroprevalência e as sorovariedades da leptospirose em seres humanos e animais no Caribe de 1979 a 2013.

**Métodos.:**

Estudo retrospectivo de revisão da literatura científica realizado sobre a epidemiologia geral, registros históricos e localizações geográficas de casos e surtos de leptospirose no Caribe no período de 1979 a 2013. As fontes primárias de informação foram identificadas através das bases de dados PubMed Central, Google Scholar, CAREC, CaribVET e Faculdade de Medicina Veterinária da Universidade das Índias Ocidentais. Os seguintes termos de busca foram usados: “human leptospirosis”, “animal leptospirosis”, “serovars”, “livestock”, “seroprevalence”, “Caribbean countries”, “risk factors”, “confirmed cases”, “suspected cases”, “MAT” e “ELISA” (leptospirose humana, leptospirose animal, sorovariedades, gado, soroprevalência, países do Caribe, fatores de risco, casos confirmados, casos suspeitos, MAT e ELISA). Os casos suspeitos e confirmados de leptospirose humana e animal foram identificados por exames laboratoriais.

**Resultados.:**

A maioria dos casos de leptospirose ocorreu no período de chuvas (junho a dezembro), apresentando uma correlação positiva com a ocorrência de enchentes. A doença foi mais prevalente no sexo masculino que no feminino devido à exposição comportamental e ocupacional. As maiores taxas de incidência de leptospirose humana foram registradas em Barbados, Trinidad e Tobago e Jamaica. Entre os animais, foi verificada a ocorrência de leptospirose em roedores, gado e cães em vários países do Caribe. Vigilância ativa insatisfatória e diagnóstico incorreto dos casos de leptospirose humana são fatores que contribuem para a subnotificação da doença.

**Conclusões.:**

Este estudo de revisão destacou a epidemiologia e a distribuição da leptospirose no Caribe. As taxas de prevalência e sorovariedades variam muito entre os países. A leptospirose representa um importante risco à saúde dos seres humanos e animais no Caribe, sendo necessário um enfoque multissetorial baseado no conceito de saúde única (one health) para reduzir a incidência da doença e proteger os indivíduos em risco. Faz-se necessário melhorar a capacidade laboratorial de identificar os casos de leptospirose, juntamente com campanhas de conscientização dirigidas ao público em geral e aos profissionais de saúde humana e animal.

Leptospirosis is a globally re-emerging zoonotic disease, caused by the pathogenic spirochete bacteria leptospira. It is common in tropical and sub-tropical areas, such as the Caribbean, where there is significant rainfall, with rodents acting as the main reservoir host and source for human leptospirosis (1). Worldwide, there are an estimated 1.03 million cases per year, resulting in 2.9 million Disability Adjusted Life Years, with the highest burden falling on resource-poor tropical countries (2). In the Caribbean, leptospirosis affects not only humans (3), but also numerous species of animals, including bats, cattle, dogs, pigs, mongoose, and opossums (4). Recent studies have indicated that Barbados, Jamaica, and Trinidad and Tobago are countries with endemic leptospirosis and the highest annual incidence in humans (10, 7.8, and 12 cases per 100 000, respectively) in the world after Seychelles (5).

In the Caribbean, leptospirosis was first reported in Puerto Rico in 1918, based on clinical findings (6). The disease was then identified in Trinidad in 1931, Barbados in 1939, and Jamaica in 1953 (7). Leptospirosis has since been identified in most countries of the Caribbean (8).

Wild animals, mainly rodents, serve as the main source or reservoir for leptospirosis (3). The *leptospira* organism can be found in the genital tract and renal tubule of animals and is excreted into the environment. The bacteria can survive up to several months in the environment (water or soil) depending on the pH (neutral or slightly alkaline) and humidity (4).

Humans can contract the disease by direct or indirect contact with the urine of infected animals or by consumption of contaminated water. Individuals living in rural communities are at a greater risk for becoming infected with leptospirosis, due to their close interaction with potentially infected animals such as rodents (9, 10). Climatic conditions (11), high rainfall, and floods (12) are elements that escalate the dissemination of leptospirosis, causing severe epidemics. Lastly, humans are more likely to contract leptospirosis when there are poor sanitary conditions and inadequate drainage (13).

Leptospirosis is overlooked and neglected largely because it affects populations living in poverty (14). The testing methods commonly used are Enzyme-Linked Immunosorbent Assay (ELISA) and the Microscopic Agglutination Test (MAT), considered the “Gold Standard” (3). Leptospirosis incidence and prevalence data are lacking due to inadequate laboratory diagnostic capacity and weak surveillance systems (15). Therefore, the prevalence of leptospirosis in the Caribbean is unknown, but many of its countries are affected.

The clinical presentation of leptospirosis is broad, and as a result, physicians may misdiagnose it as dengue, influenza, malaria, meningoencephalitis, typhoid fever, or yellow fever (16). The modified Faine’s criteria can be used to diagnose leptospirosis based on clinical symptoms, particularly when laboratory confirmation is not available (3, 17).

Human vaccines for leptospirosis are available in some countries, and clinical trials have been conducted in China, Cuba, and Russia (18). Animal leptospiral vaccines are immunogenic and safe and can reduce infection, clinical signs, and mortality. However, the vaccines available for humans and animals generally consist of one or two serovars and offer serovar-specific protection (3, 19). This is an important consideration because the distribution of strains varies in different geographic areas and populations (20).

Antibiotics commonly used for leptospirosis are penicillin and doxycycline (21). Penicillin is recommended for more severe cases, while doxycycline is recommended for less severe. Doxycycline can also be used for chemoprophylaxis when required (3, 22).

This study aims to present a brief review on the presence of leptospirosis in humans and various animal species documented in the Caribbean, including information on common reservoirs, risk factors for transmission, and serovar distribution. Furthermore, due to the large number and wide breadth of papers on leptospirosis in the Caribbean, a selection was made of those that were published most recently using robust serovar identification methods. This is not to be considered a systematic review.

## MATERIALS AND METHODS

A literature review was conducted on the general epidemiology, historical records, and geographical locations of leptospirosis outbreaks and cases in the Caribbean from 1979 – 2013. Peer-reviewed articles and reports in English or Spanish on human and/or animal leptospirosis in the Caribbean were retrieved from PubMed Central (U.S. National Library of Medicine, Bethesda, Maryland, United States), Google Scholar (Google Inc., Mountain View, California, United States), CAREC (the Caribbean Epidemiology Center; Port of Spain, Trinidad and Tobago), CaribVET (French Agricultural Research Centre for International Development, Montpellier, France), and The School of Veterinary Medicine of the University of the West Indies (Mona, Jamaica). Search terms used were: “human leptospirosis,” “animal leptospirosis,” “serovars,” “livestock,” “seroprevalence,” “Caribbean countries,” “risk factors,” “confirmed cases,” “suspected cases,” “MAT,” and “ELISA.”

## RESULTS

### Animal leptospirosis

Table 1 provides an illustration of the distribution of leptospirosis serovars found in the Caribbean.

#### Rodents.

A study conducted in Barbados (23) over two 6-month periods (October 1986 – March 1987 and October 1994 – March 1995) presented on the country’s most common serovars. Using the MAT at a titre of ≥ 100 and leptospire isolation, the rodents examined revealed that 19% and 16% harbored leptospirosa, respectively. In October 1986 – March 1987, leptospires were isolated from 12 of 63 rats (19%), with serovars Copenhageni (XI) and Arborea (I) identified. Twenty-six (41%) of these rats were Rattus rattus and 37 (59%) were R. norvegicus.

A second study (24) was conducted in October 1994 – March 1995. Leptospires were isolated from 16 of 100 rats, with serovars Copenhageni (IX), Arborea (V), and Bim (I) detected. Twenty-four (24%) and 76 (76%) of these rats were R. rattus and R. norvegicus, respectively. In Barbados, mice (Mus musculus) also have been found to carry both the serovars Arborea and Bim. Using the MAT at a titre of ≥ 100 and leptospire isolation, the mice examined revealed that 28.2% (24/85) harbored leptospirosa.

Several studies have identified leptospirosis in rats in Grenada (25), Trinidad (26, 27), Guadeloupe (28), and Jamaica (29). The majority of serovars identified were from the serogroup Icterohaemorrhagiae. The serovar Copenhageni was isolated in Grenada, Jamaica, and Trinidad; Bogvere in Guadeloupe; and Mankarso in Trinidad. The serogroup Cynopteri was also identified in Grenada. Not all papers specified the species of rat from which isolates came.

#### Dogs.

A study conducted in 2006 in Trinidad and Tobago (30) examined 419 canine sera samples using the MAT. Of the total, 160 (95 vaccinated and 65 unvaccinated) were from veterinary clinics across the island, 47 were hunting dogs, 49 were farm dogs, 113 were strays, and 50 were suspected cases of leptospirosis. Titres of 100 to < 800 were counted as evidence of previous exposure and titres ≥ 800 suggested acute/current infection. The overall seroprevalence indicated 61 (14.6%) positive for agglutinins; 23 (5.5%) positive for mixed infection; and 16 (3.8%) actively infected. Serovars Mankarso (47.5%), Autumnalis (41.0%), Icterohaemorrhagiae (32.8%), and Copenhageni (16.4%) were the most prevalent serovars in the 61 seropositive dogs (30). Adesiyun and colleagues more recently noted that Copenhageni was the predominant serovar found in dogs (27).

**TABLE 1.** The most common Leptospira serogroups/serovars identified in animals in the Caribbean, 1979 – 2013

Country	Serogroups/serovars
Barbados	– Australis/unspecified (canines) – Autumnalis/Bim (rats, mice) – Ballum/Arborea (rats, mice) – Icterohaemorrhagiae/Copenhageni (rats) – Icterohaemorrhagiae/unspecified (canines)
Cayman Islands	– Australis/Bratislava (canines) – Autumnalis/Autumnalis (canines) – Icterohaemorrhagiae/Icterohaemorrhagiae (canines)
Dominica	– Autumnalis/unspecified (cattle, goats, sheep) – Cynopteri/unspecified (cattle, goats, sheep) – Grippotyphosa/unspecified (goats, sheep) – Icterohaemorrhagiae/unspecified (cattle, goats, sheep) – Sejroe/unspecified (cattle)
Grenada	– Autumnalis/unspecified – Cynopteri/unspecified (rats) – Hebdomadis/unspecified – Icterohaemorrhagiae/Copenhageni (rats) – Icterohaemorrhagiae/Icterohaemorrhagiae (rats) – Icterohaemorrhagiae/unspecified – Icterohaemorrhagiae/Copenhageni – Icterohaemorrhagiae/Mankarso – Mini/unspecified – Pyrogenes/Pyrogenes – Sejroe/unspecified
Guadeloupe	– Icterohaemorrhagiae/Icterohaemorrhagiae (rats)
Jamaica	– Australis/Bratislava (pigs) – Canicola/canicola (cattle) – Icterohaemorrhagiae/Copenhageni (rats) – Icterohaemorrhagiae/Icterohaemorrhagiae (rats) – Sejroe/Hardjo (cattle)
Martinique	– Autumnalis/unspecified – Cynopteri/unspecified – Grippotyphosa/unspecified – Icterohaemorrhagiae/unspecified – Sejroe/unspecified
Puerto Rico	– Andamana/Andamana (canines), Icterohaemorrhagiae/Icterohaemorrhagiae (canines), Pyrogenes/Pyrogenes (canines)
Saint Vincent	– Autumnalis/unspecified (cattle, goats, sheep) – Cynopteri/unspecified (cattle, goats, sheep) – Grippotyphosa/unspecified (goats, sheep) – Icterohaemorrhagiae/unspecified (cattle, goats, sheep) – Sejroe/unspecified (cattle) – Autumnalis/autumnalis (canines) – Hebdomadis/unspecified
Trinidad and Tobago	– Icterohaemorrhagiae/Copenhageni (canines, rats) – Icterohaemorrhagiae/Icterohaemorrhagiae (canines) – Icterohaemorrhagiae/Mankarso (canines) – Panama/Panama

***Source:*** Prepared by the authors from study data.

In Barbados (31), 75% (46/78) of suspected canine cases and 62% (48/78) of unwanted dogs tested positive for leptospirosis at a MAT titre of ≥ 100. Icterohaemorrhagiae (36%) and Australis (13%) were the most prevalent serogroups.

In Puerto Rico (32), 73 (62.9%) of 116 stray dogs tested positive for leptospirosis using MAT. Fifty-three (72.6%) of the positive cases were due to the serotype Icterohaemorrhagiae, with Andamana (8.2%) and Pyrogenes (4.2%) also identified.

In Grenada (33), 20 and 67 of 105 dogs were identified as positive for leptospirosis using MAT and ELISA, respectively. The most common serovars noted were Copenhageni, Mankarso, Icterohaemorrhagiae, and Pyrogenes.

In the Cayman Island (29), one case of canine leptospirosis was reported in 2006 and another in 2010. These were laboratory confirmed and treated. In the 2010 case, there was seroconversion for L. icterohaemorrhagiae (1:1600), L. autumnalis (1:200), and L. bratislava (1:3200). Neither the testing technique nor characteristics of the dogs were mentioned in this report.

#### Livestock.

In a survey conducted in 1985 in Grenada and Trinidad, 1 206 livestock sera (cattle, pigs, sheep, goats, horses, donkeys, and chickens) were tested with MAT (34). A total of 376 of the collected sera were positive, 25% for Grenada and 44% for Trinidad. The most common serogroups found in animals in Grenada were Autumnalis, Icterohaemorrhagiae, Hebdomadis and related serogroups Sejroe, Mini, and Pyrogenes; whereas in Trinidad, those found were Icterohaemorrhagiae, Autumnalis, Hebdomadis, and Panama.

In Jamaica, a study conducted in 2011 (35) using MAT found that of 160 cattle and 19 pigs, 10% and 21%, respectively, were positive for leptospirosis. Canicola and Hardjo were the most common serovars in cattle; Bratislava was most common in pigs.

A study of 13 Caribbean countries in the Lesser Antilles (18) examined 1 788 animals—767 cattle, 579 goats, and 442 sheep—using the MAT. Of the actively-infected animals, 11 were from Martinique and 1 sheep each from St. Vincent and Dominica, with the highest seroprevalence being among cattle and goats. The most common serogroups among cattle were Sejroe, Autumnalis, Icterohaemorrhagiae, and Cynopteri; among goats, Autumnalis, Grippotyphosa, Cynopteri, and Icterohaemorrhagiae; and in sheep, Grippotyphosa and Cynopteri, followed by Icterohaemorrhagiae and Autumnalis.

### Human leptospirosis in the Caribbean – country examples

Human leptospirosis cases can be found throughout the Caribbean, including in Barbados, Cuba, Guadeloupe, Guyana, Jamaica, St. Lucia, Suriname, and Trinidad and Tobago, as shown in Table 2. The following data comes from CAREC, one of five Caribbean Regional Health Institutes that have been combined to form a single agency known as The Caribbean Public Health Agency (Port of Spain, Trinidad and Tobago).

**TABLE 2.** Annual cases of leptospirosis for all CAREC^a^ Member Countries, 2007 – 2009, and by individual country, 2010 and 2011

Year	Total cases	Bahamas	Barbados	British Virgin Islands	Dominica	Grenada	Guyana	Jamaica	St. Kitts and Nevis	St. Lucia	Suriname	St. Vincent and the Grenadines
2007	344	—^b^	—	—	—	—	—	—	—	—	—	—
2008	527	—	—	—	—	—	—	—	—	—	—	—
2009	489	—	—	—	—	—	—	—	—	—	—	—
2010	323	1	12	0	10	1	57	185	8	17	6	26
2011	415	3	44	1	29	11	153	93	12	15	25	29

***Source:*** The Caribbean Epidemiology Center (Port of Spain, Trinidad and Tobago), Annual Report 2011, Appendix 2, page 35 and Appendix 4, page 41.

a The Caribbean Epidemiology Center.

b No individual country data available.

In the 25 year period from 1980 – 2005, a total of 12 475 cases of leptospirosis were reported by Caribbean countries. Of that total, Jamaica accounted for 47%; Trinidad and Tobago, 19%; Suriname, 19%; and Barbados, 6%. The highest number of reported cases of leptospirosis (1 314 cases) occurred in 2005 due to outbreaks in Guyana and Jamaica. Nonetheless, only 148 cases were laboratory confirmed in the CAREC report. During the last 10 of that 25 year period (1995 – 2005), there were just 616 confirmed cases from CAREC Member States, representing 5% of the total cases from 1980 – 2005 in the Caribbean countries (36).

In 2006, there were a total of 308 confirmed cases reported by CAREC Member States (37). During the period from 2007 – 2011, there were 2 098 confirmed cases of leptospirosis reported by the CAREC Member Countries, as shown in Table 3 (38).

**FIGURE 1.**
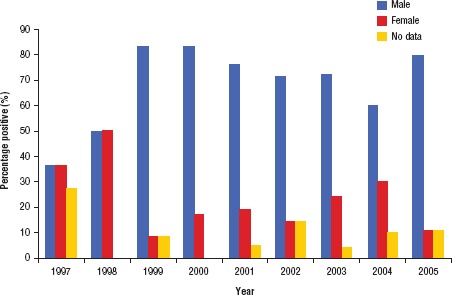
Distribution by sex of 452 laboratory-confirmed cases of human leptospirosis using Immunoglobulin M enzyme-linked immunosorbent assay on sera from 14 Caribbean countries, 1997 – 2005

***Source:*** Adesiyun et al. Human leptospirosis in the Caribbean, 1997-2005: characteristics and serotyping of clinical samples from 14 countries, 2011. Reproduced with permission.

A laboratory-based study (39) examined the prevalence of leptospirosis cases during the period from 1997 – 2005 in 14 countries across the Caribbean. The samples, having been originally sent to CAREC and tested using IGM ELISA, were serotyped using MAT. A total of 3 455 samples were submitted in 1997 – 2005; of these, 452 were seropositive for leptospirosis. Male patients (72.1%) had a significantly higher prevalence rate than female patients (19.7%) (Figure 1), and there were more cases of leptospirosis during the rainy season (June – December). The most prominent serovars observed in the study were Copenhageni (70%), Icterohaemorrhagiae (67%), and Mankarso (29%).

#### Barbados.

A survey conducted in Barbados (40) noted that the most prevalent serogroups identified in humans were Autumnalis (72%), Icterohaemorrhagiae (20%), Ballum (6%), Canicola (1%) and Grippotyphosa (1%). Bennett and Everard (41) found that sugarcane workers, garbage loaders, and drain cleaners had the highest morbidity rates for the disease.

#### Cuba.

In 2005, heavy rainfalls in October – December resulted in two outbreaks of leptospirosis in Cuba (42). A total of 293 individuals suspected of having leptospirosis were examined. The number of laboratory-confirmed cases of leptospirosis in the two outbreaks were 39/104 and 97/189, respectively. The most common serovars found were Canicola, Ballum, Icterohaemorrhagiae, and Pomona.

#### Dominica.

A study in Dominica (43) reported an outbreak in June 2010 – December 2011. A total of 40 confirmed cases of leptospirosis were noted, along with four fatalities. The majority of those infected were males whose occupations were farming or construction. Four cases were found to be infected with the serotype Icterohaemorrhagiae.

**TABLE 3.** Serogroups/serovars of leptospirosis identified in humans in the Caribbean, 1979 – 2013

Country	Serogroups/serovars
Barbados	– Autumnalis/unspecified – Ballum/unspecified – Canicola/unspecified – Grippotyphosa/unspecified – Icterohaemorrhagiae/Copenhageni and unspecified – Panama/unspecified
Cuba	– Ballum/Ballum – Canicola/Canicola – Icterohaemorrhagiae/Icterohaemorrhagiae – Pomona/Pomona
Guadeloupe	– Ballum/Arborea and Castellonis – Icterohaemorrhagiae/Icterohaemorrhagiae
Guyana	– Australis/Bratislava – Mini/Georgia – Icterohaemorrhagiae/Mankarso – Icterohaemorrhagiae/Icterohaemorrhagiae
Jamaica	– Pyrogenes/Abramis – Autumnalis/Autumnalis – Ballum/Ballum, Bataviae/Bataviae – Canicola/Canicola – Hebdomadis/Hebdomadis – Icterohaemorrhagiae/Icterohaemorrhagiae – Hebdomadis/Jules – Pomona/Pomona
Trinidad and Tobago	– Australis/Rachmati – Autumnalis/Autumnalis – Canicola/unspecified – Grippotyphosa – Icterohaemorrhagiae/Icterohaemorrhagiae, Copenhageni, and unspecified – Pyrogenes/unspecified

***Source:*** Prepared by the authors from study data.

#### Guadeloupe.

A study in 2003 – 2004 analyzed samples from a tertiary hospital in Guadeloupe to identify serovars and correlative factors to the severity of leptospirosis (28). The laboratory methods used were MAT and an in-house enzyme immune assay. A total of 168 hospitalized patients were diagnosed with leptospirosis; 132 had specific antibodies and 36 cases were confirmed by culture. Of 40 patients whose tests were positive, 18 (45%) had serovar Icterohaemorrhagiae. Also, 35% of the case-patients had serovars Arborea and Castellonis, both of which are mainly found in Guadeloupe. The serovar Icterohaemorrhagiae was found to be associated with moresevere cases than were Arborea and Castellonis.

#### Guyana.

An outbreak of leptospirosis was reported during a flood in Guyana in 2005 (22). A total of 236 suspected patients were admitted. Of these, 105 were tested with Dip-S-Tick IgM ELISA, which resulted in 52 being found positive, 41 negative, and 12 indeterminate. The majority of suspected cases were female (57%) with a median age of 32. Additional testing was completed on the 105 patients indicating 2 confirmed cases, 53 probable cases, and 50 suspected cases of leptospirosis. The most common serovars represented in 12 samples were Icterohaemorrhagiae (XI), Mankarso (XI), Georgia (X), and Bratislava (VIII).

#### Jamaica.

The results from a study in Jamaica in 2010 retrospectively reviewed cases of confirmed leptospirosis from June 2005 (when there was increased flooding) – May 2006 and concluded that males with outdoor occupations, such as farming and construction, were at a higher risk of contracting the disease (44). The confirmed serovars were Abramis, Autumnalis, Bataviae, Canicola, Hebdomadis, Icterohaemorrhagiae, Jules, and Pomona. It was also mentioned that the rainy season had contributed to the rise in cases in Jamaica and the Caribbean as a whole.

#### Martinique.

During a recreational activity on 16 May 2009 in the tropical forest of Martinique, three of the 230 participants fell ill. They were hospitalized with febrile illness, elevated liver enzyme levels, and acute renal failure, and were confirmed to be positive for leptospirosis using Polymerase Chain Reaction (PCR). Another 10 athletes later tested positive for leptospirosis by PCR or serology (45).

#### St. Lucia.

A study reported an outbreak of leptospirosis in St. Lucia in July 2010 – December 2011, due to flooding from Hurricane Thomas that resulted in 43 cases and 6 deaths (43). Increased rainfall was noted as the contributing factor for the outbreak.

#### Trinidad and Tobago.

In 2013, James and colleagues compared the seroprevalence of leptospirosis between veterinary students and other university students in Trinidad (46). The ELISA test results indicated that 11 of the 113 veterinary students were positive for leptospirosis, with an additional 19 in the borderline area. Of the 99 non-veterinary students, only 1 was positive and 12 were in the borderline area. Close contact with pets and livestock, specifically livestock farming, were found to be risk factors for veterinary students. The most prevalent serovars in veterinary students were Icterohaemorrhagiae and Copenhageni, followed by Sejroe Saxkoebing, Sejroe Sejroe, Ballum Ballum, and Bataviae Bataviae. Among non-veterinary students, Icterohaemorrhagiae Australis Rachmati serovar was the most prevalent, followed by Icterohaemorrhagiae Copenhageni and Icterohaemorrhagiae Icterohaemorrhagiae.

### Vaccination

Vaccines are available for use in cattle, dogs, and pigs. These vaccines offer serovar-specific protection from leptospirosis on a short-term basis, approximately 1 year (1). For livestock, multiple vaccines exist against various serovars and are available for those most commonly found in each country. For example, a pentavalent vaccine is available for the serovars Pomona, Grippotyphosa, Canicola, Icterohaemorrhagiae, and Hardjo in Canada and the United States (47).

There are currently two vaccines available for dogs, one containing the serovars Icterohaemorrhagiae and Canicola, and the other, Icterohaemorrhagiae, Canicola, Grippotyphosa, and Pomona (48). Information on vaccines for humans is limited and only a few commercial human vaccines exist (1). One such vaccine is produced in Cuba for use in at-risk populations and provides protection from the serovars Canicola, Copenhageni, and Mozdok (49).

## DISCUSSION

After reviewing various articles, it is evident that leptospirosis is a highly prevalent infectious disease in both animals and humans in the Caribbean. Overall incidence rates of leptospirosis in humans show fluctuations between years (38) and higher rates of infection during the rainy season, particularly in flood conditions (1). Incidence rates of leptospirosis infection from 14 Caribbean countries were higher among those 1 – 20 years of age, those 31 – 40 years of age, and in males (39). Such results can be seen in Jamaica (44) and in Trinidad and Tobago (15); but in Guyana (22), females represented the majority (57%) of suspected cases during an outbreak that followed flooding in 2005. Nevertheless, it was noted that occupations predominantly held by males, i.e. livestock farming and construction work (43), had higher morbidity rates in the Caribbean.

Animal leptospirosis in the Caribbean has been identified in a large proportion of dogs, as well as rodents and livestock. Leptospiral bacteria have been isolated from healthy and acutely infected dogs in multiple countries of the Caribbean (29, 31–33), with one study in Trinidad finding age to be a risk factor (30). For livestock, no correlations were found between age, sex, and seropositivity for leptospirosis in the Caribbean (34, 35, 50).

### Limitations

One of the limitations of this analysis was that specific serovars can only be identified by isolating the leptospire or PCR (3). Most of these studies used serological techniques, such as MAT or ELISA, that are at risk of cross-reaction, and therefore, their results should be interpreted with caution. Moreover, not all of the papers provided the specific serovar present nor the species of animal from which it was isolated. Petrakovsky and colleagues (8) also noted that studies with isolation and identification of leptospira were concentrated to Trinidad and Tobago, and that these results may not be extrapolated to the rest of the Caribbean area (8).

### Recommendations

As a zoonotic disease, effective leptospirosis surveillance, prevention, and control require a “One Heath” approach that involves related public health, animal health, and environment agencies. A collaborative system integrating animal and public health should be implemented to improve surveillance and identify serovars in order to trace human infections back to the animal source. Given that rodents are an important reservoir for leptospirosis, vector control and education are imperative to curbing this disease (3, 27, 29).

An integrated strategy should be developed and adopted to reduce and prevent the spread of leptospirosis throughout the Caribbean. Such a strategy should include: public education campaigns that warn of the possible sources of exposure; training of specific target audiences, i.e., farmers, veterinarians, and other at-risk groups; improving workplace hygiene for high-risk occupations; implementing rodent control and integrated pest management programs; flood prevention and management; improving diagnosis and case management in humans; and improving surveillance in both animals and humans.

Regarding leptospirosis in humans, there is a lack of data in the Caribbean. This can be largely attributed to a deficiency in clinical suspicion, as well as a shortage of diagnostic laboratory capacity. To reduce the risk of under and misdiagnosis, medical professionals in the Caribbean should become familiar with the modified Faine’s criteria when conducting a differential diagnosis on patients with acute undifferentiated fever. First suggested in 2004 and amended in 2012, the modified Faine’s provides a diagnostic algorithm for diagnosis based on clinical symptoms when rapid laboratory confirmation is not available (17). Part A is based on clinical presentation; Part B on epidemiological factors; Part C on bacteriological and laboratory findings; and the total score of all parts determines whether a diagnosis of leptospirosis should be made or not.

The World Health Organization has recommended initiating doxycycline therapy when leptospirosis is suspected, even if laboratory confirmation is not possible (3). Furthermore, in situations with a potential for a leptospirosis outbreak, doxycycline can be administered to at-risk individuals as a chemoprophylactic measure, as was done in Guyana in 2005 (22).

Hygienic practices, including protective clothing and footwear, avoiding contact with contaminated water, and rodent control can prevent infection. Due to the risk of leptospirosis infection (45), information should raise awareness among those who partake in freshwater recreational activities, such as swimming, in areas where the disease is known to be present. Tourists should be advised of the risk of swimming or wading in fresh, unchlorinated water.

Vaccines for leptospirosis are available for both humans and animals and can provide a certain degree of protection. Since they are serovar-specific, it is important that vaccines be aligned with the serovars circulating in the surrounding area (3, 20). In order to select the appropriate vaccines, it is essential to conduct MAT testing to identify the most prevalent serovars found in a country and among specific animal species. Underscoring this is the example of Trinidad where the two vaccines available for dogs contain the serovars Icterohaemorrhagiae, Canicola, Grippotyphosa, and Pomona (26), despite previous studies indicating that Icterohaemorrhagiae, Mankarso, Copenhageni, and Autumnalis are the most prevalent (27, 30).

Regarding laboratory capacity, its shortfalls are not only a problem for diagnosis, but also for effective surveillance. Since few of the laboratories in the Caribbean can conduct the MAT, this absence of serovar classification impedes efforts to identify the main sources of infection (3). Implementing methods to collect more accurate data on the number of cases, serovars detected, and geographical distribution would allow trace-back of the source to its animal origin, facilitating the design and improving the effectiveness of prevention and control strategies.

### Conclusions

In most Caribbean countries, leptospirosis is an endemic zoonotic disease that is under-reported. Because the disease results from environmental and occupational exposure, a “One Health” multisectoral approach is recommended for surveillance, prevention, and control. The Caribbean is one of the areas most affected by climate change. The increased temperatures, tropical storms, and humidity caused by climate change will probably drive up the number of leptospirosis cases and outbreaks. Caribbean countries need to strengthen their capacity for surveillance, diagnosis, case management, and public awareness for early detection of leptospirosis cases and trends and reducing risk of transmission to humans. Furthermore, accurate knowledge of the serovars present in each country is required to ensure effective vaccination.

At this point in time, the top priorities in the Caribbean should be increased laboratory capacity and rolling out campaigns to educate the public on how to reduce the risk of exposure to leptospirosis. When these shortcomings are addressed, the number of leptospirosis cases in humans and animals could be significantly reduced.

### Disclaimer.

Authors hold sole responsibility for the views expressed in the manuscript, which may not necessarily reflect the opinion or policy of the *RPSP/PAJPH* and/or PAHO.
